# How Does the Optimism of Students Learning a Foreign Language Affect Their Creative Self-Efficacy? The Mediating Effects of Hope and Empathy

**DOI:** 10.3389/fpsyg.2022.831593

**Published:** 2022-03-16

**Authors:** Fei Lei, Lin Lei

**Affiliations:** ^1^School of Foreign Studies, South China Normal University, Guangzhou, China; ^2^Center for Language Cognition and Assessment, South China Normal University, Guangzhou, China; ^3^School of Management, Guangdong University of Technology, Guangzhou, China

**Keywords:** optimism, hope, empathy, creative self-efficacy, creativity, foreign-language learners, positive psychology

## Abstract

Creative self-efficacy (CSE) is a core influencer of creative behavior and has a positive impact on well-being and development. However, the positive psychological processes that help to promote CSE in foreign-language learning (FLL) remain under-studied. Focusing specifically on FLL students, the present study examined the associations among optimism, hope, empathy, and CSE and investigated the possible mediating roles of hope and empathy in the relationship between optimism and CSE. A sample of 330 FLL students from two Chinese universities participated in this study. The results showed that (i) optimism, hope, and empathy were all positively related to CSE and that (ii) optimism did not directly predict CSE but indirectly and positively predicted CSE through hope and empathy. These findings suggest that optimism, empathy, and hope potentially play positive roles in facilitating CSE in FLL students. Based on the present results, some practical approaches are discussed that could help improve the CSE of FLL students, paying particular attention to the effects that potentially motivate their positivity.

## Introduction

### Creativity in Students Learning a Foreign Language

Creativity plays a key role in the process of learning a foreign language (FL), and successful FL learners (i.e., bilingual and multilingual learners) have to be highly creative because they must switch frequently between languages, and use reasoning skills and flexible thinking to make connections between the abstract and the symbolic (Cummins, 1976; Adesope et al., 2010) and combine acquired expressions in creative ways (Chomsky, 2002). Moreover, as a tool to increase cultural sustainability, creativity enhances cognitive flexibility and enables FL learners to better understand different cultures and values, thus contributing to improved competence in intercultural communication (Portnova et al., 2020). In recent years, creative learning activities—represented by creative writing and group problem-solving tasks—have been increasingly promoted in modern FL teaching (Tin, 2010; McDonough et al., 2015), thereby requiring FL learners to use their imagination and creative thinking. Therefore, it is necessary to focus more on creativity in FL learning (FLL).

It has been shown that emotions are key factors in FLL success (Li et al., 2020). Positive emotions can make FL learners more willing to absorb new language knowledge, whereas negative emotions can reduce their attention and motivation to input language (MacIntyre and Gregersen, 2012). In addition, for learners with more-positive emotions, their FLL is better because they are more attentive and more aware of language input during class (Dewaele and Alfawzan, 2018). It is evident that positive psychological traits may play a greater role in FLL than in other disciplines (MacIntyre et al., 2019).

Currently, the focus of research on the emotions of FL learners is shifting from negative to positive emotions (Dewaele, 2002; Pan and Zhang, 2021). However, the link between positive emotional feelings and FLL is still unclear (Wang et al., 2021), and the important role of positive emotions in building personal resources for FL learners requires further research (Chen and Padilla, 2019). For instance, research evidence in the field of second-language acquisition on emotion regulation and loving pedagogy is scant, yet these psychological variables contribute to successful teaching and may be a motivator for FLL (Ghanizadeh and Moafian, 2010; Pavelescu and Petric, 2018; Bielak and Mystkowska-Wiertelak, 2020; Wang et al., 2021). Furthermore, little is known about the impact of emotional influences on positive nonverbal outcomes (e.g., creativity) in FL learners (Chen and Padilla, 2019). In addition, FLL is a form of intercultural communication, and the nature of communication can be seen as the transfer of information and feelings (Zhang, 2020), making FLL more susceptible to personal emotions. Therefore, we select multiple positive emotions of FL learners as the main objects of this study.

As an important indicator of creativity, creative self-efficacy (CSE) is defined as “the belief [that] one has the ability to produce creative outcomes” (Tierney and Farmer, 2002, p. 1138). CSE refers to an individual’s perception of their ability to use creative thinking and creative functioning while problem-solving (Tierney and Farmer, 2011; Zhang et al., 2019). CSE has a strong effect on different forms of creativity (Beghetto et al., 2011), has a more substantial predictive effect than other predictors (Hülsheger et al., 2009), and can directly determine creative behavior (Tierney and Farmer, 2011). Furthermore, CSE can also increase creative production, improve well-being, reduce the negative effects of learning disabilities, and enhance professional competences and personal development (Chen, 2020; Alvarez-Huerta et al., 2021; Chang et al., 2021; Smith, 2021). It is evident that CSE has a clear effect on individual competence and emotions, but more research is needed to understand fully the mechanisms by which CSE is formed in different occupations and populations (e.g., FL learners).

Previous studies have shown that CSE is co-influenced by many factors, such as openness to experience (Shaw et al., 2021), divergent thinking skills (Azmi et al., 2018), performance orientation (Ghafoor et al., 2011), and achievement goals (Du et al., 2020). Previous research has focused on the impacts of personality and motivation on CSE, but the relationship between positive emotions and CSE is yet to been explored fully (Thundiyil et al., 2016). Moreover, previous research on the creativity of FL learners has focused on the effect of language proficiency on creativity (Stephan, 2017; Fürst and Grin, 2018) and the impact of creativity on FL output (Novikova et al., 2020; Fernandez-Fontecha, 2021), but research is lacking on the psychological factors that influence the creativity of FL learners (Chen et al., 2019). In recent years, the role of positive emotions in FLL has attracted an increasing amount of scholarly attention (MacIntyre and Gregersen, 2012; MacIntyre and Mercer, 2014; Chen et al., 2021a). The emergence of a series of theories—such as the broaden-and-build theory (Fredrickson, 2001), the control-value theory (Pekrun, 2006), and the L2 self-system theory (Dörnyei, 2005)—emphasizes the critical role of emotion in individual performance and mental health (Li, 2020), which provides theoretical guidance for emotion research. Among the different positive emotions that affect the creativity of FL learners, optimism is a variable worthy of in-depth investigation.

Several relational studies have established a positive link between optimism and CSE (Hsu et al., 2011; Zhang et al., 2019) and have shown that optimism could impact creativity through CSE. For instance, the survey by Li and Wu (2011) revealed that optimism positively predicted CSE and innovative behavior and that CSE mediated the relationship between optimism and creativity. Zhang et al. (2019) obtained similar results when they found that optimism indirectly affects social creativity through CSE. However, although previous studies have identified some important associations, they focused only on the direct effect of optimism and CSE and neglected to ask whether there are intermediary variables between these two factors. Moreover, few studies have analyzed specifically the relationship between optimism and creativity among FL learners. To fill this gap, the present study was aimed at determining which factors might affect the relationship between the optimism and CSE of FLL students.

As an essential perception of goals and the future (Snyder, 2002), hope has become a research object of great concern (Dewaele et al., 2019; MacIntyre et al., 2019), especially during the COVID-19 pandemic (Hu et al., 2021). The broaden-and-build theory argues that positive emotions (e.g., hope) may help build social and psychological resources (Fredrickson, 2001). For instance, Feng and Yin (2021) found that hope can reduce stress in medical workers and protect them from depression. Snyder (2002) argued that hope is a positive motivational state that allows individuals to realize their personal goals. Hope ultimately plays a positive role in regulating psychological states and personal growth, and because of this, another purpose of the present study was to explore the possible effect of hope on the association between optimism and CSE in FLL students.

Empathy is a basic emotion-related construct that has been the focus of much research in medicine (Hojat et al., 2015; Andersen et al., 2020) and business (Dees, 2012; Albouy and Décaudin, 2018). In recent years, research on empathy has begun to emerge within the study of FLL. For example, O’Brien (2017) suggested that multilingual learners tend to demonstrate enhanced empathy and open-mindedness, and Crosbie (2014) found that second-language learners may have higher levels of empathy for cultural diversity. However, those studies focused only on the beneficial effects of FL learning on the development of empathy, which means that the effect of empathy on the agency of FL learners remains to be understood fully (Yalcin-Tilfarlioglu and Arikan, 2012).

Empathy is also believed to play an important role in forming emotions, perceived desirability, and intentions (Miller et al., 2012; Hockerts, 2017) and so it can be inferred that optimism, hope, and CSE are intrinsically linked to empathy as affective, desire, and intention variables, respectively. Therefore, the third purpose of the present study was to investigate the roles of empathy in the association among optimism, hope, and CSE in FLL students.

Notably, enjoyment has been widely studied in research on the positive emotions of FL learners (e.g., Jiang and Dewaele, 2019; Lee, 2020). However, other positive emotion variables, such as optimism, hope, and empathy, also have important effects on learning engagement and FL performance (Khodarahmi and Zarrinabadi, 2016; Jiang and Gao, 2020; Yin, 2021), but there is a lack of research on these emotions in FLL. In addition, it has been demonstrated that there is a positive correlation among many positive psychological factors in FL learners (Chen et al., 2019), but their interactions still need to be clarified further (Chen and Padilla, 2019). Given the important role of creativity in FLL (Portnova et al., 2020), the present study takes positive emotions (optimism, hope, empathy) and CSE as the object of study and aims to present the interrelationship among these positive psychological factors.

### Optimism and CSE

Optimism has been defined as a person’s overall expectation that good results or events will occur in the future (Youssef and Luthans, 2007; Zhang et al., 2019). Individuals with an optimistic attitude tend to explain positive events in terms of personal, permanent, and pervasive causes (Seligman, 1998; Youssef and Luthans, 2007). Optimism has been valued by scholars because of its role in psychological regulation, self-control, and self-efficacy (Mäkikangas and Kinnunen, 2003; Karademas, 2006). Several studies have offered evidence of these roles and shown that individuals with an optimistic and happy mindset are more capable of controlling negative behaviors (Zhang et al., 2019), more flexible in dealing with problems, and more confident in their ability to generate creative ideas (Hirt et al., 2008).

For FL learners, optimism stimulates the development of creative ideas by allowing them to experience greater personal growth as they continually seek new ways to solve problems. For instance, the COVID-19 epidemic made traditional classroom-based FL teaching unfeasible, and many schools were forced to shift toward online instruction (Alfadda and Mahdi, 2021). In this context, being optimistic may manifest as having the confidence to develop effective online-learning methods and work hard while maintaining a sense of hope. However, despite the wealth of literature that explores the effects of optimism on creative behavior (e.g., Hsu et al., 2011; Li and Wu, 2011), few researchers have examined the optimism of FL learners and their beliefs about using creative approaches to learning.

### Hope as a Mediator

#### Optimism and Hope

Hope is described as a form of goal-directed thinking that reflects positive expectations of possible future events (Snyder et al., 1997; Roth and Hammelstein, 2007). According to Snyder’s hope model, hope consists of two elements: motivation to achieve desired goals (agency) and tracks to goal achievement (pathways; Snyder et al., 1991, 1997; Snyder, 2000; Roth and Hammelstein, 2007). As these two components illustrate, the willingness and means associated with goal achievement are the two core qualities of hope. Recently, some studies have found that hope is related to a series of positive psychological resources, including gratitude (Feng and Yin, 2021), courage (Bockorny and Youssef-Morgan, 2019), resilience (Wu et al., 2021), and optimism (Ratinen and Uusiautti, 2020), among which optimism is an important influencing factor. Highly optimistic individuals have more positive emotions and are more motivated to solve problems, which makes them more hopeful (Creamer et al., 2009; Zhang et al., 2019).

As two elements of psychological capital (Luthans et al., 2007), hope and optimism have generally been studied as a single unit (Bockorny and Youssef-Morgan, 2019). Because of this, limited research has examined the relationship between hope and optimism. Rand (2009) found a strong correlation between hope and optimism, and Snyder (2000) indicated that hope moderates the effect of optimism on social creativity. Notably, Zhang et al. (2019) argued that hope among college students was predicted by their optimism, but this still needs to be verified with a different survey sample. On the other hand, psychological research on FLL has focused mainly on negative outcomes (e.g., anxiety; MacIntyre, 2017), and little research has been conducted on the positive traits of FL learners. Exploring the role that optimism plays in building hope could shed light on the psychological process of positive emotion formation and enhance the competence and well-being of FL learners (Dewaele et al., 2019).

#### Hope and CSE

Perceived efficacy is a judgment of competence (Bandura, 2006), and many studies have shown the positive correlation between perceived efficacy and hope (Demirtas, 2019; Anderson and Feldman, 2020; Zeinalipour, 2021). As an imperative construct of perceived efficacy, CSE is concerned with an individual’s belief in their ability to produce creative outcomes. However, relatively little research has explored the relationship between CSE and hope (Yang et al., 2020). Zhang et al. (2019) found that CSE can positively predict hope, but does hope also predict CSE? This hypothesis still needs to be verified through additional empirical studies. It has been suggested that hope could improve cognitive flexibility, which in turn might help individuals creatively discover multiple ways to achieve a goal (Yang et al., 2020). It is inferred that hope may facilitate the improvement of CSE.

#### Optimism, Hope, and CSE

A few in-depth studies found that hope can act as a mediator for creativity. According to Yang et al. (2020), the socioeconomic status of a student’s family predicts the student’s creative ideation through the mediating role of hope. Furthermore, CSE has been shown to act as a mediator between optimism and hope (Zhang et al., 2019). However, hope’s mediating role in the relationship between optimism and CSE has yet to be verified. As the research overviewed thus far has shown, optimism has an effect on both hope and CSE and hope has an effect on CSE. Therefore, we postulate that optimism can directly influence the CSE of FL learners and also indirectly facilitate their CSE through the leverage of hope.

### Empathy as a Mediator

#### Optimism and Empathy

Empathy is generally defined as an affective trait and/or a cognitive ability that helps individuals experience the emotions of others and comprehend their feelings (Hogan, 1969; Bryant, 1982). Previous research found that empathy was influenced by internal factors such as prior experiences (Batson et al., 1997), educational levels (Davis, 1994), expressiveness (Carlozzi et al., 1995), values of humanism and benevolence (Stephan et al., 2011), and self-regulation skills (Rademacher and Koglin, 2020). Among the diverse internal factors, the effects of emotion and personality on empathy have received scholarly attention. According to the empathy model due to Decety and Jackson (2004), the resurgence of experienced feelings facilitates the emergence of empathy. While reviewing the evolution of empathy over 20 years, Hackney (1978) argued that qualities intrinsic to an individual’s personality can have a large influence on the sensitivity that enables them to empathize. These theories suggest that possessing an optimistic personality may be an antecedent to empathy (i.e., that optimism can increase empathy).

Numerous studies have confirmed the predictive effect of empathy on optimism in both theory and practice (Conoley et al., 2015; Hicks and Fine, 2017; Hu et al., 2021), but the effect of optimism on empathy remains to be clarified. A few empirical studies have demonstrated the predictive role of optimism on empathy. For example, in a study of 35 patients, Hoffart and Sexton (2002) found that physician empathy predicted patient optimism and that increased patient optimism elevated the level of physician empathy. Nevertheless, the sample size of 35 patients allows for idiosyncrasies (Hoffart and Sexton, 2002), so these findings need to be validated with a larger sample and different subjects. Furthermore, a study of 265 medical students by Hojat et al. (2015) revealed that optimism and empathy (two positive personality constructs) are interrelated and that one can improve with the other, although the causal link between the two is uncertain. Therefore, the association between optimism and empathy must be further verified in different settings, such as in an FLL context.

#### Empathy and CSE

It has been noted that both empathy and CSE are personality traits associated with positive characteristics and positive beliefs (Joireman et al., 2006; Dewaele et al., 2019). Therefore, it is probable that the two are correlated, but regrettably the impact of empathy on CSE has received insufficient attention. Previous research has noted that empathic individuals have positive states, such as the sense of well-being (Morelli et al., 2015) and joy (Miller et al., 2012), and are more likely to engage in harmonious in-group cooperation (de Waal, 2009). It is evident that positive emotions and favorable surroundings (relationship strength) may foster improved CSE. Furthermore, according to Hu et al. (2021), as individuals empathize with others, regulatory strategies related to perspective-taking can facilitate the exploration of possible avenues for positive change and the search for pathways to achieve those changes. In the process of continuous reflection and exploration, the ability of innovation tends to be enhanced. The above findings provide further evidence of a possible correlation between empathy and CSE.

#### Hope and Empathy

The mechanism of how hope affects empathy requires further clarification, but a positive relationship between the two has been supported theoretically. Hu et al. (2021) suggested that empathy and hope overlap because they both contribute to the broadening of collective goals and perspectives. Moreover, the concept of empathy can be extended beyond the fundamental “trying to imagine” to the “will to do something,” indicating that empathy is based on hope (Dixie et al., 1999, p. 12). However, empirical research related to hope and empathy is still in its infancy. A few studies have shown that empathy not only promotes the positive effect of hope in reducing stress but also moderates the effect of hope on post-stress growth (Hu et al., 2021). Studies in the medical field have also found a positive correlation between the level of hope and perceived maternal empathy in sick children (Lloyd et al., 2009). The above findings reveal a potential correlation between hope and empathy.

#### Optimism, Empathy, and CSE

Several studies have provided empirical evidence for the relationship among optimism, empathy, and CSE. For instance, the survey by Ji et al. (2018) of 214 children found that empathy was significantly and positively related to optimism and creativity, respectively. Dionigi et al. (2020) found that empathy, optimism, and self-efficacy in health workers were all predictive of psychological well-being, which suggests that well-being has a commonality (or “share variance” in statistical terms; Hojat et al., 2015) with these three variables. These studies suggest that optimism, hope, and empathy may be correlated.

#### Optimism, Hope, Empathy, and CSE

Established theories support the correlation among optimism, hope, empathy, and CSE. Hu et al. (2021) noted that optimism and hope are two positive emotions, while empathy is a fundamental response to emotion. Through empirical studies, Conoley et al. (2015) found that high levels of CSE constitute a psychological force, which can be expressed through empathy. As the studies mentioned in this literature review have revealed, hope has a potential predictive effect on empathy (Dixie et al., 1999; Lloyd et al., 2009). From these theories, it can be inferred that hope and empathy may play a chain mediating role between optimism and CSE.

## Research Aims and Hypotheses

As mentioned above, previous research has shown pairwise relationships between CSE and (i) optimism, (ii) hope, and (iii) empathy. However, we do not have a complete description of how these variables are interrelated in one system, especially for FLL students. Identifying the associations among these variables in the same system is meaningful because it allows knowing how multiple affective variables frequently experienced in the learning process jointly influence FL learners’ CSE, and to further understand the mechanism of synergistic influence of psychological variables on creativity, thus providing theoretical support for FL teaching. In addition, the clarification of the relationships among these variables can also draw FL teachers’ attention to students’ learning status and guide them to use psychological regulation to enhance FL students’ learning effectiveness and creativity, rather than only training creativity at the level of language and thinking.

**Figure 1:**
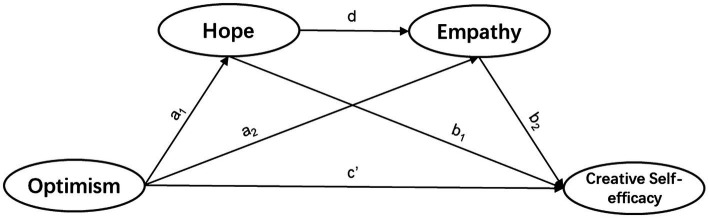
Conceptual mediation model regarding mediating effects of hope and empathy on relationship between optimism and creative self-efficacy (CSE).

The present study aims to explore how positive psychological factors (i.e., optimism, hope, and empathy) predict CSE in FL learners. Furthermore, this research will extend previous research by considering the possible mediating roles of hope and empathy in the relationship between optimism and CSE. The mediating roles of hope and empathy are important because if hope and empathy mediate the association between optimism and CSE, then they may offer possible ways to reduce the negative impact of low optimism on CSE (Zhang et al., 2019). Figure 1 illustrates the conceptual mediation model regarding the mediating effects of hope and empathy on the relationship between optimism and CSE. Therefore, we propose the following five hypotheses.

*Hypothesis 1*: The optimism, hope, empathy, and CSE of FLL students are positively related to each other.

*Hypothesis 2*: The optimism of FLL students can positively predict their CSE.

*Hypothesis 3*: The optimism of FLL students can predict their CSE through the mediator of hope.

*Hypothesis 4*: The optimism of FLL students can predict their CSE through the mediator of empathy.

*Hypothesis 5*: The optimism of FLL students can predict their CSE through the chain mediating effect of hope and empathy.

## Methods

### Participants and Procedures

The survey respondents consisted of 330 FLL students from two major universities in southern China, of which 69 were male and 261 were female. In terms of FL background, all the respondents had studied English as their first FL (L2), of which 219 had studied a second FL (L3) and 25 had learned three or more FLs (L4, L5). The respondents came from different disciplines, including English literature (193 respondents, 58.5%), e-commerce (62 respondents, 18.8%), Chinese literature (36 respondents, 10.9%), and other fields (39 respondents, 11.8%). With respect to academic levels, third-year undergraduates (153) and fourth-year undergraduates (113) accounted for 80.6% of the sample; graduate students (31), sophomores (18), and freshmen (15) also participated in the survey. The average age of the respondents was 21.28 years.

The COVID-19 epidemic meant that the questionnaire was administered online. To avoid self-selection bias in online investigation, we explained the importance of the survey to the respondents before the questionnaires were distributed. To encourage the respondents to take the survey, we promised to share the survey results with them so that they might better understand their emotional and psychological traits. To avoid the possible negative backwash effects of the respondents’ final exam (Ahmad and Rao, 2012), we conducted the questionnaire two weeks before the end of the course.

The questionnaire was distributed with the assistance of eight professors who asked their students to use their free time to complete it. We chose WJX^1^ as the platform to host the questionnaire; this platform is a popular online survey website in China, and it is highly stable and easy to operate. To ensure that the respondents did not omit any items and thus to avoid missing values, all items in the questionnaire were set as mandatory otherwise it could not be submitted.

Participants were asked to complete the online questionnaire within 6 days of reading an informed-consent form to learn about the research purpose and decide whether to participate in the survey voluntarily. The questionnaire was conducted anonymously to protect the respondents’ privacy. The survey system showed that the respondents took an average of 6 min to complete the questionnaire at an average of 3.8 s per item, which is within the expected time frame.

### Instruments

#### Measurement of Optimism

We used the Life Orientation Test (LOT) due to Scheier and Carver (1985) to measure optimism in FLL students. The LOT is a widely used standardized measure of optimism (Luthans et al., 2005; Hojat et al., 2015) that is answered on a five-point Likert scale (1 = “Strongly disagree” to 5 = “Strongly agree”). The original scale includes 12 Likert items, but following the principle of parsimony, we favored using a smaller set of items in the scale (Li and Wu, 2011). Four filler items (e.g., “It’s important for me to keep busy”) and four reversed items (e.g., “I hardly ever expect things to go my way”) were removed, and we ended up keeping four positive items (e.g., “In uncertain times, I usually expect the best”). The statistics showed that the streamlined scale had high internal consistency (*α* = 0.72) and conformed to standard structural validity [*χ*^2^/df = 2.38; CFI = 0.99; TLI = 0.97; RMSEA (90% CI) = 0.06 (0.00–0.15); SRMR = 0.02].

#### Measurement of Hope

To measure the level of hope in FLL students, we used the State Hope Scale (SHS) developed by Snyder et al. (1996). The scale consists of six items and includes two dimensions: agency thinking (e.g., “Right now, I see myself as being pretty successful”) and pathway thinking (e.g., “I can think of many ways to reach my current goals”). All items were rated on a five-point Likert scale (1 = “Strongly disagree” to 5 = “Strongly agree”). The SHS has been used widely in numerous countries including China and has shown good reliability and validity (Luthans et al., 2007; Zhou et al., 2017; Feng and Yin, 2021). The reliability test showed that the internal consistency was satisfactory (*α* = 0.79 for total scale, *α* = 0.65 for agency thinking, and *α* = 0.68 for pathway thinking). Confirmatory factor analysis (CFA) with two factors indicated good fit indices for the questionnaire [*χ*^2^/df = 3.21, CFI = 0.97, TLI = 0.94, RMSEA (90% CI) = 0.08 (0.05–0.12), SRMR = 0.03].

#### Measurement of Empathy

To measure empathy in FLL students, we used the Online Simulation (OS) subscale of the Questionnaire of Cognitive and Affective Empathy (QCAE) revised by Wang and Su (2019). The original version of the QCAE was developed by Reniers et al. (2011) and has been used widely in psychology, behavioral sciences, education, and medicine (Myszkowski et al., 2017; Andersen et al., 2020; Zirenko et al., 2021). Wang and Su (2019) revised the QCAE twice according to the characteristics of the Chinese adolescent population, and the modified scale showed satisfactory internal consistency (*α* = 0.80). Given that cognitive empathy develops more consistently during adolescence than does emotional empathy, the OS subscale, which measures cognitive empathy effectively (Wang and Su, 2019), was chosen for the present study. The OS subscale consisted of three items (e.g., “I sometimes try to understand my friends better by imagining how things look from their perspective”) scored on a five-point Likert scale (1 = “Strongly disagree” to 5 = “Strongly agree”). The internal reliability of the scale was acceptable (*α* = 0.65), and excellent fit indices were derived from CFA [*χ*^2^/df = 0.00, CFI = 1.00, TLI = 1.00, RMSEA (90% CI) = 0.00 (0.00–0.00), SRMR = 0.00].

#### Measurement of Creative Self-Efficacy

The Creative Self-efficacy Scale (Tierney and Farmer, 2002) was used to measure creativity in FL learners with four items (e.g., “I have confidence in my ability to solve problems creatively”). This scale has been used in various fields including business, economics, and education (Gong et al., 2009; Puente-Díaz, 2016; Hora et al., 2021) and has shown good psychometric properties in different countries (Liu et al., 2017; Alvarez-Huerta et al., 2021). This scale can measure the following three aspects of FLL students: (i) whether they have confidence in overcoming difficulties encountered in the process of FLL by finding innovative methods; (ii) whether they are conscious of absorbing the good learning experiences of others so as to create their own FLL approaches; and (iii) whether they can constantly come up with new ways to meet the challenges in FLL. The students indicated the extent to which they believed that each statement described how they felt about their creative ability (Gong et al., 2009). All items were rated on a five-point Likert scale (1 = “Strongly disagree” to 5 = “Strongly agree”) and demonstrated a good level of reliability (*α* = 0.79), and CFA indicated a satisfactory fit [*χ*^2^/df = 1.40, CFI = 0.99, TLI = 0.99, RMSEA (90% CI) = 0.04 (0.00–0.12), SRMR = 0.01].

#### Control Variable

Gender, which has been shown to influence CSE (Karwowski, 2011; Yang et al., 2020), is considered to be a vital control variable and was included in the data analyses (male was coded as 1, female was coded as 2). Karwowski (2011) found that men tend to perceive their creativity as higher, while women underestimate their CSE. One reason for this difference may be that women lack confidence in their intelligence (Furnham et al., 1999), whereas men are more confident in their creative self-perceptions and self-concept in problem-solving (Khanam and Sen, 1998; Marsh et al., 2006). In addition, increased creativity requires greater tolerance of ambiguity (Zenasni et al., 2008), whereas women’s traditional identities give them greater “other-directedness” that diminishes this tolerance (Taylor, 2011; Karwowski et al., 2013). This suggests the need to control for possible confounding effects of gender when examining how psychological structures may affect CSE.

### Data Analysis

We used the SPSS software (version 26.0) for statistical analysis. The reported descriptive data included the means (M) and standard deviations (SD) of respondents’ scores on optimism, hope, empathy, and CSE (see Table 1). Pearson’s bivariate correlation analysis was performed to examine the associations between variables. To measure the strength of correlation coefficients, the values of 0.25 (weak), 0.40 (moderate), and 0.60 (strong) were used as reference standards (Plonsky and Oswald, 2014).

**Table 1:** Mean (M), standard deviation (SD), skewness, and kurtosis of all variables as their bivariate Pearson intercorrelations (*N* = 330).

	*M*	SD	Skewness	Kurtosis	1	2	3	4	5
1. Opt	3.55	0.72	−0.58	0.43	1				
2. Hope	3.47	0.55	−0.54	2.13	0.75^**^	1			
3. Emp	3.94	0.56	−0.23	0.02	0.27^**^	0.38^**^	1		
4. CSE	3.39	0.62	−0.11	0.62	0.42^**^	0.54^**^	0.36^**^	1	
5. Gender	1.79	0.41	–	–	−0.01	−0.06	0.05	−0.14^*^	1

* *p* *< 0.05;*

** *p < 0.01*.

*Opt, optimism; Emp, empathy; CSE, creative self-efficacy; Gender: 1 = male, 2 = female*.

To determine the statistical method used, we examined the assumption of normality by calculating skewness and kurtosis for optimism, hope, empathy, and CSE. Using the criteria of Ghasemi and Zahediasl (2012), if the sample size is more significant than 200 and the absolute Z-standardized values of skewness and kurtosis are less than 2.58, then the data follow a normal distribution.

To test for mediating effects, we used the PROCESS v3.5 Model 6 macro for SPSS developed by Hayes (2018) (Model 6 is the model with two mediating variables) with 5,000 random-sample bootstrapping confidence intervals (CIs; Hayes, 2013; Hu et al., 2021). Furthermore, to measure the strength of indirect effects, three values of indirect effect-size estimates were used as criteria (Kenny, 2020): 0.01 (weak), 0.09 (moderate), and 0.25 (strong).

## Results

### Preliminary Analyses

The results of the descriptive statistics are presented in Table 1. The CSE score (mean = 3.39) was close to 3.4 (five-point scale), and the empathy score (mean = 3.94) even approached 4 (five-point scale), indicating that Chinese FLL students have positive psychological traits. Moreover, the scores for optimism and hope were greater than 3.4 (five-point scale), suggesting that this population maintained positive emotions.

As shown in Table 1, the absolute values of skewness ranged from 0.11 to 0.58, and the absolute values of kurtosis ranged from 0.02 to 2.13, which when taken together illustrated acceptable normality of the study variables (Ghasemi and Zahediasl, 2012; Hinton, 2014; Wen et al., 2018). Thus, all variables could be used for correlation and regression analysis (Chen et al., 2021b).

The results of the correlation analysis in Table 1 illustrate that as predicted, optimism and hope were strongly positively correlated (*r* = 0.75, *p* < 0.01). CSE was moderately positively correlated with optimism and hope, separately (*r* = 0.42–0.54, all *p* < 0.01), and empathy was weakly positively correlated with optimism, hope, and CSE, separately (*r* = 0.27–0.38, all *p* < 0.01). These results are consistent with hypothesis 1 and provided the preconditions for the validity of hypotheses 2–5. Gender was also found to be weakly correlated with CSE as a control variable (*r* = −0.14, *p* < 0.05).

### Test of Mediation

Controlling for gender, we tested the mediating effects of hope and empathy between optimism and CSE, which yielded the following results (see Table 2 for all path coefficients and Table 3 for direct and indirect effects).

**Table 2:** Standardized regression coefficients and standard errors in mediation model (gender was statistically controlled in all models).

Predictor variables		Outcome variables										
		Hope				Empathy				Creative self-efficacy		
		*β*	SE	*t*		*β*	SE	t		*β*	SE	*t*
Optimism	*a*_1_	0.56	0.03	16.80^**^	*a*_2_	−0.04	0.06	−0.65	*c*′	0.05	0.06	0.87
Hope					*d*	0.42	0.07	5.88^**^	*b*_1_	0.47	0.07	6.47^**^
Empathy									*b*_2_	0.21	0.05	3.95^**^
*R* ^2^				0.46				0.15				0.34

** *p < 0.01*.

**Table 3:** Direct and indirect effects of optimism on CSE through hope and empathy (three pathways for indirect effects).

	Estimated	SE	95% CI (lower)	95% CI (upper)	Relative effect
Total effect	0.36^a^	0.05	0.27	0.45	–
Direct effect	0.05	0.06	−0.06	0.16	–
Indirect effects	0.31^a^	0.06	0.21	0.42	86.03%
*Path 1:* Opt→Hope→CSE	0.27^a^	0.06	0.17	0.38	74.26%
*Path 2:* Opt→Hope→Emp → CSE	0.05^a^	0.02	0.02	0.09	14.04%
*Path 3:* Opt→Emp → CSE	−0.01	0.01	−0.04	0.02	−2.27%

a *Empirical 95% confidence interval (CI) does not overlap with zero*.

*Opt, optimism; Emp, empathy; CSE, creative self-efficacy; SE, standardized error*.

The direct path from optimism to CSE was not significant (*β* = 0.05, *t* = 0.87, *p* = 0.38 > 0.05), nor was the direct effect of optimism on CSE (effect = 0.05, SE = 0.06, 95% CI = −0.06–0.16), which show that hypothesis 2 was not established. Moreover, the total indirect effect was 0.31 (SE = 0.06, 95% CI = 0.21–0.42), and the ratio of the total indirect effect (0.31) to the total effect (0.36) was 86.03%, indicating that optimism mainly affected CSE through mediating variables (i.e., hope and empathy).

Specifically, the direct path from optimism to hope was significant (*β* = 0.56, *t* = 16.80, *p* = 0.00 < 0.01), as was the direct path from hope to CSE (*β* = 0.47, *t* = 6.47, *p* = 0.00 < 0.01). Furthermore, the indirect path (path 1) from optimism to CSE through hope was significant, with an indirect effect of 0.27 (SE = 0.06, 95% CI = 0.17–0.38). The above results suggest that a mediating effect of hope existed between optimism and CSE, which verifies hypothesis 3.

However, the direct path from optimism to empathy was not significant (*β* = −0.04, *t* = −0.65, *p* = 0.52 > 0.05), nor was the indirect path (path 3) from optimism to CSE through empathy (effect = −0.01, SE = 0.01, 90% CI = −0.04–0.02), and the above results disprove hypothesis 4.

**Figure 2:**
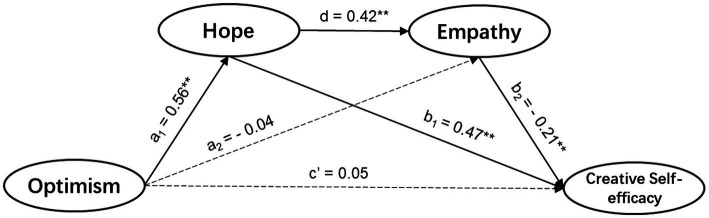
Path diagram of modified mediation model linking optimism and CSE through hope and empathy. A dashed line suggests a non-significant relationship between the two variables, while a solid line suggests a significant relationship. All the path coefficients were standardized. *N* = 330. ^**^p < 0.01.

Additionally, the path from empathy to CSE was significant (*β* = 0.21, *t* = 3.95, *p* = 0.00 < 0.01), as was the direct path from hope to empathy (*β* = 0.42, *t* = 5.88, *p* = 0.00 < 0.01). Moreover, the indirect path (path 2) from optimism to CSE through both hope and empathy was significant, and the indirect effect was 0.05 (SE = 0.02, 95% CI = 0.02–0.09). The above results show that optimism might influence CSE through the mediating effects of hope and empathy, which validates hypothesis 5. The modified model is shown in Figure 2.

As suggested by Kenny (2020), the total indirect effect (0.31) could be considered very large, while the indirect effect of path 1 (0.27) was large, and the indirect effect of path 2 (0.05) was small to medium. In total, the overall mediation model could explain 34% of the variance in CSE. This study also found that gender, as a control variable, could affect CSE (*β* = −0.19, *t* = −2.70, *p* = 0.01 < 0.05), which is a finding that is consistent with existing studies (Beghetto, 2006; Karwowski, 2011).

## Discussion

### Personal Variables and CSE in FL Learners

The descriptive results of this study suggest that all four personal factors (optimism, hope, empathy, and CSE) of the FLL students were related to each other (hypothesis 1), and in particular, optimism, hope, and empathy were positively correlated with CSE to a moderate degree (*r* = 0.36–0.54), which is consistent with previous studies (Hojat et al., 2015; Zhang et al., 2019; Ratinen and Uusiautti, 2020; Yang et al., 2020; Hu et al., 2021). In addition, regression analyses demonstrated the positive predictive effects of optimism on hope, hope on empathy, hope on CSE, and empathy on CSE.

As shown in the correlation analysis in Table 1, gender was associated with CSE, but the relationship was considerably weaker than the one that emerged between the three psychological variables (i.e., optimism, hope, and empathy) and CSE. This finding indicates that males exhibited slightly higher levels of CSE than did females in this sample of FLL students. This result is consistent with previous studies, which showed that gender was correlated with beliefs about creativity (Karwowski, 2011; Yang et al., 2020). Thus, gender has been controlled in the mediation analysis.

The results do not support hypothesis 2, indicating that optimism was not a direct predictor of CSE but could indirectly affect CSE through mediating variables. This result is consistent with the findings of Hsu et al. (2011) in the Taiwanese service industry, where optimism had an indirect effect on employees’ creativity. These results extend the findings of Li and Wu (2011) and Zhang et al. (2019) on the relationship between optimism and CSE by considering two possible mediating variables (hope and empathy). Therefore, the direct effect of optimism on CSE may be reduced when taking hope and empathy into account in the model.

A possible reason for the above results may be that happy emotions have been shown to make people more playful and exploratory, leading them to take novel and adventurous approaches to solving problems (Bless et al., 1990). Optimism and hope are both happy emotions, but hope seems to be more emotional than is optimism (Peterson and Seligman, 2004). Moreover, hopeful individuals may give up on short-term emotional goals; instead, they look to the future and choose flexible and effective strategies to achieve long-term goals (Trope et al., 2003; Hirt et al., 2008; Lopez et al., 2009). This could explain why hope affects CSE more directly than does optimism. On the other hand, individuals in happy moods are more focused on emotional regulation and the feelings of others; they explore possible avenues for mood change and engage in human-centered design from the perspective of others, which could promote cognitive improvement and constitute a positive effect of empathy on creativity (Hirt et al., 2008; So and Joo, 2017).

Nevertheless, the effect of optimism on CSE is certain, regardless of the direct or indirect impact. For FLL students, the need to master a new language outside its context often presents difficulties and challenges (Khodarahmi and Zarrinabadi, 2016). Therefore, maintaining positive emotions (e.g., optimism and hope) in FL learning is important because students with positive emotions are more engaged in learning, better at self-regulation, more willing to optimize learning strategies, and more self-motivated, which can enhance their language proficiency, emotional competence, and creative thinking (Fredrickson, 2003; Khodarahmi and Zarrinabadi, 2016; Chen and Padilla, 2019; Yin, 2021). Moreover, FLL is often accompanied by the learning of the culture in which the target language is spoken, which involves empathy for other cultures (Kokkola et al., 2022). Students with high empathy are more tolerant, understanding, and accepting of foreign cultures, which leads them to think more deeply about different cultures and thus contributes to creativity (Savchenko et al., 2018).

### Hope and Empathy as Mediators

The primary purpose of this study was to explore the relationship between optimism and CSE by examining the mediating roles of hope and empathy. Mediation analyses revealed the mediating role of hope in the relationship between optimism and CSE (hypothesis 3), which is consistent with previous research on these constructs (Creamer et al., 2009; Rand, 2009; Zhang et al., 2019; Yang et al., 2020). Instead of giving up when faced with difficulties, optimistic individuals maintain high expectations and positive emotions for the future (Rand, 2009), which contributes to the development of creative thinking and motivates them to experiment with multiple approaches to solving problems and reaching goals (Snyder, 2002; Amabile et al., 2005; Hirt et al., 2008). Along with problem-solving, the ability to use creativity was enhanced, and the confidence in one’s capacity for innovation increased. Conversely, individuals with low optimism tended to lack self-confidence and motivation. They felt confused and hopeless about the future and could not develop practical and innovative approaches to problem resolution, thus reducing their CSE (Snyder, 2000).

In particular, note that the mediating effect of hope was strong (effect = 0.27), which suggests that optimism enhances CSE primarily through hope. Hope is an essential effector of self-efficacy (Avey et al., 2008), and hopeful individuals possess high levels of cognitive flexibility (Baas et al., 2013; Yang et al., 2020), which helps them creatively discover multiple viable ways to achieve goals. Furthermore, hope can increase intrinsic motivation and inspire individuals to make an effort to accomplish tasks (Yang et al., 2020). Finding a viable pathway usually requires stimulating creative thinking and abilities, which effectively improves initiative and agency to solve problems in a creative manner (Snyder, 2002; Shalley and Gilson, 2004).

Notably, the indirect effect of optimism on CSE through empathy was not statistically significant in this mediation model (hypothesis 4). Similarly, the direct impact of optimism on empathy was also not effective, despite the significant correlation between these two variables (*r* = 0.27). It was inferred that hope could play an adequate mediating role in the relationship between optimism and empathy. In other words, highly optimistic students may foster greater hope to cultivate higher levels of empathy. These results extend the findings of Hojat et al. (2015), Hoffart and Sexton (2002), and Rodrigue et al. (2004) about the relationship between optimism and empathy by considering a new potential mediator (hope). Accordingly, the indirect effect of optimism on CSE through empathy may be attenuated when hope is considered.

In fact, hope and empathy are strongly related. Hopeful individuals think more flexibly (Lopez et al., 2000), which makes them more open-minded and more capable to perceive the emotional information conveyed by others, thus rendering a greater degree of empathy (Carlozzi et al., 1995). In addition, as with the products of hope (e.g., life goals and beliefs), empathy may facilitate individuals to enlarge their collective goals and perspectives (Fite et al., 2014; Hu et al., 2021), thereby further amplifying their attributes required for CSE.

Furthermore, the results of mediation analyses supported the link between optimism and CSE through hope and empathy. Hope is a positive emotion and a constructive expectation of possible future events (Roth and Hammelstein, 2007). Individuals who are extremely hopeful have flexible thinking and creativity, as well as a belief in diverse pathways toward the future (Lopez et al., 2000). Evidently, hope facilitates the enhancement of problem-solving dynamics (Snyder, 2000) and promotes the formation of creative thinking (Liu, 2016), thus contributing to the completion of creative tasks (Baas et al., 2013).

On the other hand, empathy has cognitive properties. Individuals with high levels of empathy are adept at understanding the experiences, concerns, and perspectives of others and are able to communicate and cooperate effectively (Hojat, 2007; Hojat et al., 2014). Effective communication and collaboration set the stage for the collision and integration of different perspectives, which fosters the emergence of creative ideation. Moreover, empathizing with others is an exploration of emotional regulation (Hu et al., 2021) that requires individuals to engage in creative thinking while participating in constant self-reflection, which also contributes to CSE. For FL learners, reading the FL requires cognitive effort and creativity because they must engage cognitively and emotionally with fictional characters, at which point empathy can be viewed as a creative cognitive act (Kokkola et al., 2022). Therefore, it is understandable that the FLL students who were more optimistic tended to have higher levels of hope and empathy, which in turn enhanced their CSE.

Theoretically, the mediating effects found in the present study are supported by positive psychology theory and motivation theory. According to the broaden-and-build theory due to Fredrickson (2001), positive emotional experiences (i.e., optimism and hope) contribute to establishing enduring psychological and intellectual resources (i.e., empathy and CSE). In addition, the L2 self-system theory due to Dörnyei (2005) suggests that the emotions experienced when envisioning the future (optimism) facilitate the construction of the future bilingual self (hope), the enhancement of motivation to learn an FL, and the expansion of thinking patterns (CSE; MacIntyre and Gregersen, 2012). Consequently, it is evident that positive psychological traits play an important role in FLL (MacIntyre et al., 2019) and that it is essential to develop positive feelings or cognition through positive psychological activities (Chen et al., 2019).

Practically, our research findings support the implementation of positive education in FL teaching. Positive education—defined as “education in traditional skills and well-being” (Seligman et al., 2009, p. 293)—can facilitate the development of “skills for happiness” such as positive moods (Seligman et al., 2009), which leads to a broader attention span (Rowe et al., 2007), a more holistic mindset (Isen et al., 1991), and creative thinking (Estrada et al., 1994). In FL practice, positive education could be integrated with language instruction to develop learners’ positive emotions, positive personality, and positive engagement while teaching language knowledge so as to continuously improve both the FL proficiency and sense of well-being. Concretely, institutions could cultivate the optimism and positive expectations of FLL students for the future through the approaches such as “teaching positive education,” “embedding positive education,” and “living positive education” (Seligman et al., 2009, pp. 304–307) in order to engage them in more-concentrated learning, increase their appreciation of different individuals within a group, and reduce their levels of prejudice toward others (Johnson and Fredrickson, 2005), which will in turn promote more-creative thinking.

## Limitations and Suggestions

The present study had several limitations. First, the measures of CSE in FLL students should be more specific (e.g., the efficacy for creative writing in an FL or for thoughtful presentations or discussions in an FL). Second, the present study was conducted with a sample of Chinese college students, and future research might assess whether these relationships are valid for other countries and occupations. Third, including other potential psychological factors (e.g., flow and resilience) could be considered with the aim of further exploring and understanding the complex mechanisms underlying the effects of CSE.

## Conclusion and Implications

The results of the present empirical study showed that optimism, hope, empathy, and CSE among a sample of FLL students were at high levels, which indicates that this group maintained positive moods and personality traits and a willingness to engage in creative activities. Furthermore, the present study found that optimism, hope, and empathy were all positively related to CSE and that optimism could not directly influence CSE but could indirectly influence CSE through hope and empathy (i.e., hope and empathy played important roles in mediating the relationship between optimism and CSE). This suggests that effective interventions of hope or empathy may motivate low-optimism FL learners to use creative approaches for problem solving in FLL (Zhang et al., 2019).

Competencies related to creativity are considered necessary for advancing technology (Carlozzi et al., 1995), yet the creativity of FL learners is understudied, making the present paper a valuable exploration of their CSE and related influences. Whereas previous studies only tested partial relationships among factors and offered incomplete findings, our research integrated multiple factors and examined them in the same system and thus makes a novel contribution to the ongoing critical conversation. The interpretation of this four-factor model allows a deeper understanding of the associations among optimism, hope, empathy, and CSE (e.g., the mediating effects of hope and empathy) and ultimately offers a more profound comprehension of the psychological profiles of FLL students.

In summary, the three influencing factors verified in the present study (optimism, hope, and empathy) are instructive for implementing targeted strategies to cultivate creativity in FL teaching. For school administrators, teachers should be trained in the core theories of positive psychology to recognize the importance of positive psychological interventions for FLL success, thus creating an environment conducive to academic achievement for students (Wang et al., 2021). For teachers, they ought to be conscious and prepared to deal with students’ affective ambivalence (MacIntyre et al., 2009) and take care to monitor their emotions regularly (e.g., by asking students to report regularly on the status of their FLL). When negative emotions (e.g., pessimism and despair) are identified in students, teachers should be proactive in understanding the reasons affecting their emotions in a timely manner and target positive psychological interventions in the teaching process. Specifically, the following methods can be used to enhance FL students’ optimism, hope, and empathy, thus enhancing their creativity.

To enhance optimism in FLL students, teachers should be aware of FLL optimism as an important individual difference and the key role that it might play in FLL (Khodarahmi and Zarrinabadi, 2016) and encourage students to face adversities and setbacks rationally and positively. For example, COVID-19 reduced the opportunities for face-to-face training with native speakers, which led to depression among some students. To address this issue, teachers could help students to develop a positive belief that the COVID-19 pandemic will end and guide them to find alternative ways (e.g., language exchanges with foreigners through online media) to continue practicing their FLs (Hu et al., 2021). In addition, teachers should guide students to engage in positive thinking and self-motivation in FLL, and develop their emotion regulation skills and positive mindset so that they have stable emotional strength to cope with emotional fluctuations in the learning process (Li and Xu, 2019).

To make FLL students more hopeful, teachers should help students maintain their agentic control of FL learning. For instance, when English majors begin studying French (L3), they are often less motivated by the cumbersome grammar of the language. Teachers may help students build confidence and hope that they will be able to learn French well by guiding them to look for similarities between English and French, as well as finding creative ways to enhance their French learning by drawing on their knowledge and learning experience in English. Moreover, teachers should make students aware of the benefits and importance of FL mastery for their future development and help them set clear and achievable goals, thus enhancing their confidence and expectations in FLL (Li and Xu, 2019).

To develop empathy in FLL students, focus should be placed on the nurturing of students’ cooperative learning skills, which helps to improve interpersonal relationships, develop cooperative skills, establish the concept of equality and caring, promote the development of empathy, deepen the understanding of different cultures, and enhance problem-solving skills (Ning, 2013). For example, teachers could provide students with more group-based tasks, such as working together on creative writing and cultural presentations, as a way to promote interactive communication and cooperation, to cultivate their awareness of listening to, understanding, and assimilating the perspectives of others, and to enhance their interest in foreign cultures, thereby enhancing their creativity in FLs. Moreover, given that the conception of persona (i.e., using a persona during ideation) can raise empathy levels (Miaskiewicz and Kozar, 2011), teachers should give students more opportunities to role-play in FL classes because doing so helps to promote the development of empathy skills (Rivers et al., 2016).

## Data Availability Statement

The raw data supporting the conclusions of this article will be made available by the authors, without undue reservation.

## Ethics Statement

Ethical review and approval was not required for the study on human participants in accordance with the local legislation and institutional requirements. The patients/participants provided their written informed consent to participate in this study.

## Author Contributions

FL designed the research, analyzed the data, and wrote the manuscript. LL collected the data, performed the statistical analysis, and revised the manuscript. All authors contributed to the article and approved the submitted version.

## Funding

This work was supported by the National Social Science Fund of China.

## Conflict of Interest

The authors declare that the research was conducted in the absence of any commercial or financial relationships that could be construed as a potential conflict of interest.

## Publisher’s Note

All claims expressed in this article are solely those of the authors and do not necessarily represent those of their affiliated organizations, or those of the publisher, the editors and the reviewers. Any product that may be evaluated in this article, or claim that may be made by its manufacturer, is not guaranteed or endorsed by the publisher.

## Footnotes

^1^ https://www.wjx.cn

## References

[ref1] Adesope O. O. Lavin T. Thompson T. Ungerleider C. (2010). A systematic review and meta-analysis of the cognitive correlates of bilingualism. Rev. Educ. Res. 80, 207–245. doi: 10.3102/0034654310368803

[ref2] Ahmad S. Rao C. (2012). A review of the pedagogical implications of examination washback. Humanit. Soc. Sci. 2, 11–20.

[ref3] Albouy J. Décaudin J.-M. (2018). Age differences in responsiveness to shocking prosocial campaigns. J. Consum. Mark. 35, 328–339. doi: 10.1108/JCM-02-2016-1713

[ref4] Alfadda H. A. Mahdi H. S. (2021). Measuring students’ use of zoom application in language course based on the technology acceptance model (TAM). J. Psycholinguist. Res. 50, 883–900. doi: 10.1007/s10936-020-09752-1, PMID: 33398606 PMC7781650

[ref5] Alvarez-Huerta P. Muela A. Larrea I. (2021). Student engagement and creative confidence beliefs in higher education. Think. Skills Creat. 40:100821. doi: 10.1016/j.tsc.2021.100821

[ref6] Amabile T. M. Barsade S. G. Mueller J. S. Staw B. M. (2005). Affect and creativity at work. Adm. Sci. Q. 50, 367–403. doi: 10.2189/asqu.2005.50.3.367, PMID: 35073805

[ref7] Andersen F. A. Johansen A.-S. B. Sondergaard J. Andersen C. M. Assing Hvidt E. (2020). Revisiting the trajectory of medical students’ empathy, and impact of gender, specialty preferences and nationality: a systematic review. BMC Med. Educ. 20:52. doi: 10.1186/s12909-020-1964-5, PMID: 32066430 PMC7027232

[ref8] Anderson C. L. Feldman D. B. (2020). Hope and physical exercise: The contributions of Hope, self-efficacy, and optimism in accounting for variance in exercise frequency. Psychol. Rep. 123, 1145–1159. doi: 10.1177/0033294119851798, PMID: 31142190

[ref9] Avey J. B. Wernsing T. S. Luthans F. (2008). Can positive employees help positive organizational change? Impact of psychological capital and emotions on relevant attitudes and behaviors. J. Appl. Behav. Sci. 44, 48–70. doi: 10.1177/0021886307311470

[ref10] Azmi N. H. Surat S. Marzuki M. A. Yusoff A. N. Rahman S. (2018). Effects of idea generation module on Students’ creative self-efficacy. Adv. Sci. Lett. 24, 8463–8466. doi: 10.1166/asl.2018.12589

[ref11] Baas M. Roskes M. Sligte D. Nijstad B. A. De Dreu C. K. W. (2013). Personality and creativity: The dual pathway to creativity model and a research agenda. Soc. Personal. Psychol. Compass 7, 732–748. doi: 10.1111/spc3.12062

[ref12] Bandura A. (2006). “Guide for constructing self-efficacy scales,” in Self-Efficacy Beliefs of Adolescents. eds. Pajares F. Urdan T. (Greenwich, CT: Information Age), 1–43.

[ref13] Batson C. D. Early S. Salvarani G. (1997). Perspective taking: imagining how Another feels versus imaging how you would feel. Personal. Soc. Psychol. Bull. 23, 751–758. doi: 10.1177/0146167297237008, PMID: 16140345

[ref14] Beghetto R. A. (2006). Creative self-efficacy: correlates in middle and secondary students. Creat. Res. J. 18, 447–457. doi: 10.1207/s15326934crj1804_4

[ref15] Beghetto R. A. Kaufman J. C. Baxter J. (2011). Answering the unexpected questions: exploring the relationship between students’ creative self-efficacy and teacher ratings of creativity. Psychol. Aesthet. Creat. Arts 5, 342–349. doi: 10.1037/a0022834

[ref16] Bielak J. Mystkowska-Wiertelak A. (2020). Language teachers’ interpersonal learner-directed emotion-regulation strategies. Lang. Teach. Res. 1235. doi: 10.1177/1362168820912352

[ref17] Bless H. Bohner G. Schwarz N. Strack F. (1990). Mood and persuasion: A cognitive response analysis. Personal. Soc. Psychol. Bull. 16, 331–345. doi: 10.1177/0146167290162013, PMID: 12375769

[ref18] Bockorny K. Youssef-Morgan C. M. (2019). Entrepreneurs’ courage, psychological capital, and life satisfaction. Front. Psychol. 10. doi: 10.3389/fpsyg.2019.00789, PMID: 31024410 PMC6461011

[ref19] Bryant B. K. (1982). An index of empathy for children and adolescents. Child Dev. Perspect. 53, 413–425. doi: 10.2307/1128984, PMID: 34671185

[ref20] Carlozzi A. F. Bull K. S. Eells G. T. Hurlburt J. D. (1995). Empathy as related to creativity, dogmatism, and expressiveness. Aust. J. Psychol. 129, 365–373. doi: 10.1080/00223980.1995.9914974, PMID: 7650632

[ref21] Chang Y. S. Kao J. Y. Wang Y. Y. Huang S. C. (2021). Effects of cloud-based learning on student's engineering design creativity with different creative self-efficacy. Think. Skills Creat. 40:100813. doi: 10.1016/j.tsc.2021.100813

[ref22] Chen Y. (2020). Relationship between owner-Managers’ creative self-efficacy, career involvement and career subjective well-being: evidence from marine-related Enterprises in Coastal City. J. Coast. Res. 115, 442–445. doi: 10.2112/JCR-SI115-124.1

[ref23] Chen X. J. He J. B. Fan X. T. (2019). Relationships between openness to experience, cognitive flexibility, self-esteem, and creativity among bilingual college students in the US. Int. J. Biling. Educ. Biling. 25, 342–354. doi: 10.1080/13670050.2019.1688247

[ref24] Chen X. J. Padilla A. M. (2019). Emotions and creativity as predictors of resilience among L3 learners in the Chinese educational context. Curr. Psychol. 41, 406–416. doi: 10.1007/s12144-019-00581-7

[ref25] Chen X. Vallerand R. J. Padilla A. M. (2021a). On the role of passion in second language learning and flourishing. J. Happiness Stud. 22, 2761–2779. doi: 10.1007/s10902-020-00339-0

[ref26] Chen Z. Zhang P. Lin Y. Li Y. (2021b). Interactions of trait emotional intelligence, foreign language anxiety, and foreign language enjoyment in the foreign language speaking classroom. J. Multiling. Multicult. Dev. 1-21, 1–21. doi: 10.1080/01434632.2021.1890754

[ref27] Chomsky N. (2002). Syntactic Structures. Berlin: De Gruyter Mouton.

[ref28] Conoley C. W. Pontrelli M. E. Oromendia M. F. Carmen Bello B. D. Nagata C. M. (2015). Positive empathy: A therapeutic skill inspired by positive psychology. J. Clin. Psychol. 71, 575–583. doi: 10.1002/jclp.22175, PMID: 25873531

[ref29] Creamer M. O’Donnell M. L. Carboon I. Lewis V. Densley K. McFarlane A.. (2009). Evaluation of the dispositional Hope scale in injury survivors. J. Res. Pers. 43, 613–617. doi: 10.1016/j.jrp.2009.03.002

[ref30] Crosbie V. (2014). Capabilities for intercultural dialogue. Lang. Intercult. Commun. 14, 91–107. doi: 10.1080/14708477.2013.866126

[ref31] Cummins J. (1976). “The influence of bilingualism on cognitive growth: a synthesis of research findings and explanatory hypotheses. Working papers on bilingualism, no. 9”. (Toronto: The Ontario Institute for Studies in education, bilingual education project).

[ref32] Davis M.H. (1994). Empathy: A Social Psychological Approach. Boulder, Colorado: Westview Press.

[ref33] de Waal F. (2009). The Age of Empathy: Nature’s Lessons for a Kinder Society. New York: Three Rivers Press.

[ref34] Decety J. Jackson P. L. (2004). The functional architecture of human empathy. Behav. Cogn. Neurosci. Rev. 3, 71–100. doi: 10.1177/1534582304267187, PMID: 15537986

[ref35] Dees J. G. (2012). A tale of two cultures: charity, problem solving, and the future of social entrepreneurship. J. Bus. Ethics 111, 321–334. doi: 10.1007/s10551-012-1412-5

[ref36] Demirtas A. S. (2019). Secure attachment and self-efficacy in Early adolescence: The mediating role of Hope. Egitim Ve Bilim 44, 175–190. doi: 10.15390/eb.2019.8100

[ref37] Dewaele J.-M. (2002). Psychological and sociodemographic correlates of communicative anxiety in L2 and L3 production. Int. J. Biling. 6, 23–38. doi: 10.1177/13670069020060010201

[ref38] Dewaele J.-M. Alfawzan M. (2018). Does the effect of enjoyment outweigh that of anxiety in foreign language performance? Stud. Sec. Lang. Learn. Teach. 8, 21–45. doi: 10.14746/ssllt.2018.8.1.2

[ref39] Dewaele J.-M. Chen X. Padilla A. M. Lake J. (2019). The flowering of positive psychology in foreign language teaching and acquisition. Front. Psychol. 10:02128. doi: 10.3389/fpsyg.2019.02128, PMID: 31607981 PMC6769100

[ref40] Dionigi A. Casu G. Gremigni P. (2020). Associations of self-efficacy, optimism, and empathy with psychological health in healthcare volunteers. Int. J. Environ. Res. Public Health 17:66001. doi: 10.3390/ijerph17166001, PMID: 32824812 PMC7460217

[ref41] Dixie Q.H. West C. Washington J.M. (1999). The Courage to Hope: From Black Suffering to Human Redemption. Boston, USA: Beacon Press.

[ref42] Dörnyei Z. (2005). The Psychology of the Language Learner: Individual Differences in Second Language Acquisition. Mahwah: Lawrence Erlbaum.

[ref43] Du K. Wang Y. Ma X. Luo Z. Wang L. Shi B. (2020). Achievement goals and creativity: the mediating role of creative self-efficacy. Educ. Psychol. 40, 1249–1269. doi: 10.1080/01443410.2020.1806210

[ref44] Estrada C. A. Isen A. M. Young M. J. (1994). Positive affect improves creative problem solving and influences reported source of practice satisfaction in physicians. Motiv. Emot. 18, 285–299. doi: 10.1007/BF02856470

[ref45] Feng L. Yin R. (2021). Social support and Hope mediate the relationship Between gratitude and depression among front-line medical staff During the pandemic of COVID-19. Front. Psychol. 12:623873. doi: 10.3389/fpsyg.2021.623873, PMID: 33776846 PMC7987792

[ref46] Fernandez-Fontecha A. (2021). The role of learner creativity in L2 semantic fluency: an exploratory study. System 103:102658. doi: 10.1016/j.system.2021.102658

[ref47] Fite P. J. Gabrielli J. Cooley J. L. Haas S. M. Frazer A. Rubens S. L.. (2014). Hope as a moderator of the associations Between common risk factors and frequency of substance use Among Latino adolescents. J. Psychopathol. Behav. Assess. 36, 653–662. doi: 10.1007/s10862-014-9426-1, PMID: 25364098 PMC4212824

[ref48] Fredrickson B. L. (2001). The role of positive emotions in positive psychology - The broaden-and-build theory of positive emotions. Am. Psychol. 56, 218–226. doi: 10.1037/0003-066X.56.3.218, PMID: 11315248 PMC3122271

[ref49] Fredrickson B. L. (2003). The value of positive emotions. Am. Sci. 91, 330–335. doi: 10.1511/2003.4.330, PMID: 35182793

[ref50] Furnham A. Fong G. Martin N. (1999). Sex and cross-cultural differences in the estimated multifaceted intelligence quotient score for self, parents and siblings. Personal. Individ. Differ. 26, 1025–1034. doi: 10.1016/S0191-8869(98)00201-3

[ref51] Fürst G. Grin F. (2018). Multilingualism and creativity: a multivariate approach. J. Multiling. Multicult. Dev. 39, 341–355. doi: 10.1080/01434632.2017.1389948

[ref52] Ghafoor A. Qureshi T. M. Azeemi H. R. Hijazi S. T. (2011). Mediating role of creative self-efficacy. Afr. J. Bus. Manag. 5, 11093–11103. doi: 10.5897/ajbm11.876

[ref53] Ghanizadeh A. Moafian F. (2010). The role of EFL teachers’ emotional intelligence in their success. ELT J. 64, 424–435. doi: 10.1093/elt/ccp084

[ref54] Ghasemi A. Zahediasl S. (2012). Normality tests for statistical analysis: a guide for non-statisticians. Int. J. Endocrinol. Metab. 10, 486–489. doi: 10.5812/ijem.3505, PMID: 23843808 PMC3693611

[ref55] Gong Y. Huang J.-C. Farh J.-L. (2009). Employee learning orientation, transformational leadership, and employee creativity: The mediating role of employee creative self-efficacy. Acad. Manag. J. 52, 765–778. doi: 10.5465/amj.2009.43670890

[ref56] Hackney H. (1978). The evolution of empathy. Pers. Guid. J. 57, 35–38. doi: 10.1002/j.2164-4918.1978.tb05091.x, PMID: 35203902

[ref57] Hayes A. F. (2013). Introduction to mediation, moderation, and conditional process analysis: A regression-based approach. J. Educ. Meas. 51, 335–337. doi: 10.1111/jedm.12050

[ref58] Hayes A.F. (2018). Introduction to Mediation, Moderation, and Conditional Process Analysis. A Regression-Based Approach. 2nd Edn. New York: Guilford.

[ref59] Hicks D. Fine L. (2017). Physician empathy and sensitivity to a Patient's emotional journey can be critical to patient optimism and empowerment. J. Thorac. Oncol. 12:S1812. doi: 10.1016/j.jtho.2017.09.471

[ref60] Hinton P.R. (2014). Statistics Explained. 3rd Edn. New York: Routledge.

[ref61] Hirt E. R. Devers E. E. McCrea S. M. (2008). I want to be creative: exploring the role of hedonic contingency theory in the positive mood-cognitive flexibility link. J. Pers. Soc. Psychol. 94, 214–230. doi: 10.1037/0022-3514.94.2.94.2.214, PMID: 18211173

[ref62] Hockerts K. (2017). Determinants of social entrepreneurial intentions. Theor. Pract. 41, 105–130. doi: 10.1111/etap.12171, PMID: 29953517

[ref63] Hoffart A. Sexton H. (2002). The role of optimism in the process of schema-focused cognitive therapy of personality problems. Behav. Res. Ther. 40, 611–623. doi: 10.1016/S0005-7967(01)00027-4, PMID: 12051481

[ref64] Hogan R. (1969). Development of an empathy scale. J. Consult. Clin. Psychol. 33, 307–316. doi: 10.1037/h0027580, PMID: 4389335

[ref65] Hojat M. (2007). Empathy in Patient Care: Antecedents, Development, Measurement, and Outcomes. New York: Springer.

[ref66] Hojat M. Bianco J. A. Mann D. Massello D. Calabrese L. H. (2014). Overlap between empathy, teamwork and integrative approach to patient care. Med. Teach. 37, 755–758. doi: 10.3109/0142159X.2014.971722, PMID: 25314019

[ref67] Hojat M. Vergare M. Isenberg G. Cohen M. Spandorfer J. (2015). Underlying construct of empathy, optimism, and burnout in medical students. Int. J. Med. Educ. 6, 12–16. doi: 10.5116/ijme.54c3.60cd, PMID: 25633650 PMC4332366

[ref68] Hora S. Lemoine G. J. Xu N. Shalley C. E. (2021). Unlocking and closing the gender gap in creative performance: A multilevel model. J. Organ. Behav. 42, 297–312. doi: 10.1002/job.2500

[ref69] Hsu M. L. A. Hou S.-T. Fan H.-L. (2011). Creative self-efficacy and innovative behavior in a service setting: optimism as a moderator. J. Creat. Behav. 45, 258–272. doi: 10.1002/j.2162-6057.2011.tb01430.x

[ref70] Hu Y. X. Ye B. J. Im H. (2021). Hope and post-stress growth during COVID-19 pandemic: The mediating role of perceived stress and the moderating role of empathy. Personal. Individ. Differ. 178, 110831. doi: 10.1016/j.paid.2021.110831 PMC9755888 36540790

[ref71] Hülsheger U. R. Anderson N. Salgado J. F. (2009). Team-level predictors of innovation at work: A comprehensive meta-analysis spanning three decades of research. J. Appl. Psychol. 94, 1128–1145. doi: 10.1037/a0015978, PMID: 19702361

[ref72] Isen A. M. Rosenzweig A. S. Young M. J. (1991). The influence of positive affect on clinical problem solving. Med. Decis. Mak. 11, 221–227. doi: 10.1177/0272989X9101100313, PMID: 1881279

[ref73] Ji S. Park E.-H. Jo Y.-J. (2018). An analysis of relationships among Young children’ language comprehension ability, creativity. Emp. Opt. Korean Educ. Inq. 36, 221–244. doi: 10.22327/kei.2018.36.3.221

[ref74] Jiang Y. Dewaele J.-M. (2019). How unique is the foreign language classroom enjoyment and anxiety of Chinese EFL learners? System 82, 13–25. doi: 10.1016/j.system.2019.02.017

[ref75] Jiang L. Gao J. (2020). Fostering EFL Learners’ digital empathy through multimodal composing. RELC J. 51, 70–85. doi: 10.1177/0033688219898565

[ref76] Johnson K. J. Fredrickson B. L. (2005). “We All look the same to me”: positive emotions eliminate the own-race bias in face recognition. Psychol. Sci. 16, 875–881. doi: 10.1111/j.1467-9280.2005.01631.x, PMID: 16262774 PMC1808554

[ref77] Joireman J. Kamdar D. Daniels D. Duell B. (2006). Good citizens to the end? It depends: empathy and concern with future consequences moderate the impact of short-term time horizon on organizational citizenship behaviors. J. Appl. Psychol. 91, 1307–1320. doi: 10.1037/0021-9010.91.6.1307, PMID: 17100486

[ref78] Karademas E. C. (2006). Self-efficacy, social support and well-being - The mediating role of optimism. Personal. Individ. Differ. 40, 1281–1290. doi: 10.1016/j.paid.2005.10.019, PMID: 34752466

[ref79] Karwowski M. (2011). It doesn’t hurt to ask. But sometimes it hurts to believe: polish students’ creative self-efficacy and its predictors. Psychol. Aesthet. Creat. Arts 5, 154–164. doi: 10.1037/a0021427

[ref80] Karwowski M. Lebuda I. Wisniewska E. Gralewski J. (2013). Big five personality traits as the predictors of creative self-efficacy and creative personal identity: does gender matter? J. Creat. Behav. 47, 215–232. doi: 10.1002/jocb.32

[ref81] Kenny D.A. (2020). SEM: Measuring Model Fit. Available at: http://davidakenny.net/cm/fit.htm (Accessed October 2, 2021).

[ref82] Khanam M. Sen A. K. (1998). The influence of creativity, sex and type of school on creativity self-perception. Soc. Sci. Int. 14, 60–77.

[ref83] Khodarahmi E. Zarrinabadi N. (2016). Self-regulation and academic optimism in a sample of Iranian language learners: variations Across achievement group and gender. Curr. Psychol. 35, 700–710. doi: 10.1007/s12144-015-9340-z

[ref84] Kokkola L. Fjallstrom E. Rydstrom U. (2022). Creativity and cognition in fiction by teenage learners of English. Lang. Lit. 10721. doi: 10.1177/09639470211072171

[ref85] Lee J. S. (2020). The role of grit and classroom enjoyment in EFL learners’ willingness to communicate. J. Multiling. Multicult. Dev. 1–17. doi: 10.1080/01434632.2020.1746319

[ref86] Li C. (2020). The relationship between emotional intelligence and English achievement. Foreign Lang. World 1, 69–78.

[ref87] Li C. C. Dewaele J. M. Jiang G. Y. (2020). The complex relationship between classroom emotions and EFL achievement in China. Appl. Linguist. Rev. 11, 485–510. doi: 10.1515/applirev-2018-0043

[ref88] Li C.-H. Wu J.-J. (2011). The structural relationships Between optimism and innovative behavior: understanding potential antecedents and mediating effects. Creat. Res. J. 23, 119–128. doi: 10.1080/10400419.2011.571184

[ref89] Li C. Xu J. (2019). Trait emotional intelligence and classroom emotions: A positive psychology investigation and intervention Among Chinese EFL learners. Front. Psychol. 10. doi: 10.3389/fpsyg.2019.02453, PMID: 31736840 PMC6834770

[ref90] Liu L. (2016). College students’ sense of hope and its influence on creative thinking. unpublished doctoral dissertation. Shanghai, China: Shanghai Normal University.

[ref91] Liu W. Pan Y. Luo X. Wang L. Pang W. (2017). Active procrastination and creative ideation: The mediating role of creative self-efficacy. Personal. Individ. Differ. 119, 227–229. doi: 10.1016/j.paid.2017.07.033

[ref92] Lloyd S. M. Cantell M. Pacaud D. Crawford S. Dewey D. (2009). Brief report: Hope, perceived maternal empathy, medical regimen adherence, and glycemic control in adolescents with type 1 diabetes. J. Pediatr. Psychol. 34, 1025–1029. doi: 10.1093/jpepsy/jsn141, PMID: 19168503

[ref93] Lopez S. J. Floyd R. K. Ulven J. C. Snyder C. R. (2000). “Hope therapy: helping clients build a house of hope,” in Handbook of Hope: Theory, Measures, and Applications. ed. Snyder C. R. (Cambridge, MA: Academic Press), 123–150.

[ref94] Lopez S. J. Rose S. Robinson C. Marques S. C. Pais-Ribeiro J. (2009). “Measuring and promoting hope in schoolchildren,” in Handbook of Positive Psychology in the Schools. eds. Gilman R. Huebner E. S. Furlong M. J. (Mahwah, NJ: Lawrence Erlbaum), 55–68.

[ref95] Luthans F. Avolio B. J. Avey J. B. Norman S. M. (2007). Positive psychological capital: measurement and relationship with performance and satisfaction. Pers. Psychol. 60, 541–572. doi: 10.1111/j.1744-6570.2007.00083.x, PMID: 28971881

[ref96] Luthans F. Avolio B. J. Walumbwa F. O. Li W. (2005). The psychological Capital of Chinese Workers: exploring the relationship with performance. Manag. Organ. Rev. 1, 249–271. doi: 10.1111/j.1740-8784.2005.00011.x

[ref97] MacIntyre P. D. (2017). “An overview of language anxiety research and trends in its development” in New Insights into Language Anxiety: Theory, Research and Educational Implications. eds. Gkonou C. Daubney M. Dewaele J.-M. (Bristol: Multilingual Matters), 11–30.

[ref98] MacIntyre P. Gregersen T. (2012). Emotions that facilitate language learning: The positive-broadening power of the imagination. Stud. Sec. Lang. Learn. Teach. 2, 193–213. doi: 10.14746/ssllt.2012.2.2.4

[ref99] MacIntyre P. D. Gregersen T. Mercer S. (2019). Setting an agenda for positive psychology in SLA: theory, practice, and research. Mod. Lang. J. 103, 262–274. doi: 10.1111/modl.12544

[ref100] MacIntyre P. D. MacKinnon S. P. Clément R. (2009). “Embracing affective ambivalence: a research agenda for understanding the interdependent processes of language axiety and motivation” in Cultural Identity and Language Anxiety. eds. Cheng P. Yan J. X. (Guillin: Guangxi Normal University Press), 3–34.

[ref101] MacIntyre P. D. Mercer S. (2014). Introducing positive psychology to SLA. Stud. Sec. Lang. Learn. Teach. 4, 153–172. doi: 10.14746/ssllt.2014.4.2.2

[ref102] Mäkikangas A. Kinnunen U. (2003). Psychosocial work stressors and well-being: self-esteem and optimism as moderators in a one-year longitudinal sample. Personal. Individ. Differ. 35, 537–557. doi: 10.1016/S0191-8869(02)00217-9

[ref103] Marsh H. W. Trautwein U. Ludtke O. Koller O. Baumert J. (2006). Integration of multidimensional self-concept and core personality constructs: construct validation and relations to well-being and achievement. J. Pers. 74, 403–456. doi: 10.1111/j.1467-6494.2005.00380.x, PMID: 16529582

[ref104] McDonough K. Crawford W. J. Mackey A. (2015). Creativity and EFL students’ language use during a group problem-solving task. TESOL Quart. 49, 188–199. doi: 10.1002/tesq.211

[ref105] Miaskiewicz T. Kozar K. A. (2011). Personas and user-centered design: how can personas benefit product design processes? Des. Stud. 32, 417–430. doi: 10.1016/j.destud.2011.03.003

[ref106] Miller T. L. Grimes M. G. McMullen J. S. Vogus T. J. (2012). Venturing for others with heart and head: how compassion encourages social entrepreneurship. Acad. Manag. Rev. 37, 616–640. doi: 10.5465/amr.2010.0456

[ref107] Morelli S. A. Lieberman M. D. Zaki J. (2015). The emerging study of positive empathy. Soc. Personal. Psychol. Compass 9, 57–68. doi: 10.1111/spc3.12157, PMID: 34861961

[ref108] Myszkowski N. Brunet-Gouet E. Roux P. Robieux L. Malezieux A. Boujut E.. (2017). Is the questionnaire of cognitive and affective empathy measuring two or five dimensions? Evidence in a French sample. Psychiatry Res. 255, 292–296. doi: 10.1016/j.psychres.2017.05.047, PMID: 28600998

[ref109] Ning H. (2013). The impact of cooperative learning on English as a foreign language tertiary Learners’ social skills. Soc. Behav. Pers. 41, 557–567. doi: 10.2224/sbp.2013.41.4.557

[ref110] Novikova I. A. Berisha N. S. Novikov A. L. Shlyakhta D. A. (2020). Creativity and personality traits as foreign language acquisition predictors in university linguistics students. Behav. Sci. 10:35. doi: 10.3390/bs10010035, PMID: 31952184 PMC7016726

[ref111] O’Brien M.G. (2017). Literature review on the impact of second-language learning. Available at: www.acpi.ca/documents/litreview.pdf (Accessed May 15, 2020).

[ref112] Pan C. Zhang X. (2021). A longitudinal study of foreign language anxiety and enjoyment. Lang. Teach. Res. 1-24:136216882199334. doi: 10.1177/1362168821993341

[ref113] Pavelescu L. M. Petric B. (2018). Love and enjoyment in context: four case studies of adolescent EFL learners. Stud. Sec. Lang. Learn. Teach. 8, 73–101. doi: 10.14746/ssllt.2018.8.1.4

[ref114] Pekrun R. (2006). The control-value theory of achievement emotions: assumptions, corollaries, and implications for educational research and practice. Educ. Psychol. Rev. 18, 315–341. doi: 10.1007/s10648-006-9029-9

[ref115] Peterson C.E. Seligman M.E.P. (2004). Character Strengths and Virtues: A Handbook and Classification. New York, NY: Oxford University Press.

[ref116] Plonsky L. Oswald F. L. (2014). How big is “big”? Interpreting effect sizes in L2 research. Lang. Learn. 64, 878–912. doi: 10.1111/lang.12079

[ref117] Portnova T. Ortega-Martín J. L. Zurita-Ortega F. González-Valero G. (2020). The educational interrelation of narrative creativity and written expression dimensions as an innovative and didactic process in learning a foreign language. Sustain. For. 12:7274. doi: 10.3390/su12187274

[ref118] Puente-Díaz R. (2016). Creative self-efficacy: An exploration of its antecedents, consequences, and applied implications. Aust. J. Psychol. 150, 175–195. doi: 10.1080/00223980.2015.1051498, PMID: 26431487

[ref119] Rademacher A. Koglin U. (2020). Self-regulation as a mediator for the relationship Between parenting and the development of behavior problems and social-emotional competences in elementary school children. Kindheit Und Entwicklung 29, 21–29. doi: 10.1026/0942-5403/a000297

[ref120] Rand K. L. (2009). Hope and optimism: latent structures and influences on grade expectancy and academic performance. J. Pers. 77, 231–260. doi: 10.1111/j.1467-6494.2008.00544.x, PMID: 19076999

[ref121] Ratinen I. Uusiautti S. (2020). Finnish Students' knowledge of climate change mitigation and its connection to Hope. Sustain. For. 12:2181. doi: 10.3390/su12062181

[ref122] Reniers R. L. E. P. Corcoran R. Drake R. Shryane N. M. Völlm B. A. (2011). The QCAE: A questionnaire of cognitive and affective empathy. J. Pers. Assess. 93, 84–95. doi: 10.1080/00223891.2010.528484, PMID: 21184334

[ref123] Rivers A. Wickramasekera I. E. Pekala R. J. Rivers J. A. (2016). Empathic features and absorption in fantasy role-playing. Am. J. Clin. Hypn. 58, 286–294. doi: 10.1080/00029157.2015.1103696, PMID: 26675155

[ref124] Rodrigue J. R. Cornell D. L. Jackson S. I. Kanasky W. Marhefka S. Reed A. I. (2004). Are organ donation attitudes and beliefs, empathy, and life orientation related to donor registration status? Prog. Transplant. 14, 56–60. doi: 10.1177/152692480401400109, PMID: 15077739

[ref125] Roth M. Hammelstein P. (2007). Hope as an emotion of expectancy: first assessment results. Psyc. Soc. Med. 4, 1–9. PMC2736531 19742296

[ref126] Rowe G. Hirsh J. B. Anderson A. k. Smith E. E. (2007). Positive affect increases the breadth of attentional selection. Proc. Natl. Acad. Sci. 104, 383–388. doi: 10.1073/pnas.0605198104, PMID: 17182749 PMC1765470

[ref127] Savchenko E. Kharitonova E. Kovsh E. Bhatti N. (2018). “The cross-cultural and communicative competences formation in students' training process in polyethnic society.” in *12th International Technology, Education and Development Conference (INTED)*, 3333–3336.

[ref128] Scheier M. F. Carver C. S. (1985). Optimism, coping, and health: assessment and implications of generalized outcome expectancies. Health Psychol. 4, 219–247. doi: 10.1037/0278-6133.4.3.219, PMID: 4029106

[ref129] Seligman M. (1998). Learned Optimism. New York: Pocket.

[ref130] Seligman M. E. P. Ernst R. M. Gillham J. Reivich K. Linkins M. (2009). Positive education: positive psychology and classroom interventions. Oxf. Rev. Educ. 35, 293–311. doi: 10.1080/03054980902934563, PMID: 35086535

[ref131] Shalley C. E. Gilson L. L. (2004). What leaders need to know: A review of social and contextual factors that can foster or hinder creativity. Leadersh. Q. 15, 33–53. doi: 10.1016/j.leaqua.2003.12.004, PMID: 34721137

[ref132] Shaw A. Kapnek M. Morelli N. A. (2021). Measuring creative self-efficacy: An item response theory analysis of the creative self-efficacy scale. Front. Psychol. 12. doi: 10.3389/fpsyg.2021.678033, PMID: 34305732 PMC8297998

[ref133] Smith J. E. (2021). Creative self-efficacy: An essential transition skill for students With learning disabilities. Interv. Sch. Clin. 10249. doi: 10.1177/10534512211024938

[ref134] Snyder C.R. (2000). Handbook of Hope: Theory, Measures, and Applications. Cambridge, MA: Academic Press.

[ref135] Snyder C. R. (2002). Hope theory: rainbows in the mind. Psychol. Inq. 13, 249–275. doi: 10.1207/S15327965PLI1304_01

[ref136] Snyder C. R. Harris C. Anderson J. R. Holleran S. A. Irving L. M. Sigmon S. T.. (1991). The will and the ways: development and validation of an individual-differences measure of hope. J. Pers. Soc. Psychol. 60, 570–585. doi: 10.1037/0022-3514.60.4.570, PMID: 2037968

[ref137] Snyder C. R. Hoza B. Pelham W. E. Rapoff M. Ware L. Danovsky M.. (1997). The development and validation of the Children’s Hope Scale1. J. Pediatr. Psychol. 22, 399–421. doi: 10.1093/jpepsy/22.3.399, PMID: 9212556

[ref138] Snyder C. R. Sympson S. C. Ybasco F. C. Borders T. F. Babyak M. A. Higgins R. L. (1996). Development and validation of the state Hope scale. J. Pers. Soc. Psychol. 70, 321–335. doi: 10.1037/0022-3514.70.2.321, PMID: 8636885

[ref139] So C. Joo J. (2017). Does a persona improve creativity? Des. J. 20, 459–475. doi: 10.1080/14606925.2017.1319672

[ref140] Stephan E. (2017). The influence of a foreign versus native language on creativity. Creat. Res. J. 29, 426–432. doi: 10.1080/10400419.2017.1376544

[ref141] Stephan E. Liberman N. Trope Y. (2011). The effects of time perspective and level of construal on social distance. J. Exp. Soc. Psychol. 47, 397–402. doi: 10.1016/j.jesp.2010.11.001, PMID: 21836728 PMC3153444

[ref142] Taylor S. (2011). Negotiating oppositions and uncertainties: gendered conflicts in creative identity work. Fem. Psychol. 21, 354–371. doi: 10.1177/0959353510386095

[ref143] Thundiyil T. G. Chiaburu D. S. Li N. Wagner D. T. (2016). Joint effects of creative self-efficacy, positive and negative affect on creative performance. Chin. Manag. Stud. 10, 726–745. doi: 10.1108/CMS-06-2016-0126

[ref144] Tierney P. Farmer S. M. (2002). Creative self-efficacy: its potential antecedents and relationship to creative performance. Acad. Manag. J. 45, 1137–1148. doi: 10.2307/3069429

[ref145] Tierney P. Farmer S. M. (2011). Creative self-efficacy development and creative performance over time. J. Appl. Psychol. 96, 277–293. doi: 10.1037/a0020952, PMID: 20954756

[ref146] Tin T. B. (2010). Language creativity and co-emergence of form and meaning in creative writing tasks. Appl. Linguis. 32, 215–235. doi: 10.1093/applin/amq050

[ref147] Trope Y. Gervey B. Bolger N. (2003). The role of perceived control in overcoming defensive self-evaluation. J. Exp. Soc. Psychol. 39, 407–419. doi: 10.1016/S0022-1031(03)00035-0

[ref148] Wang Y. Derakhshan A. Zhang L. J. (2021). Researching and practicing positive psychology in second/foreign language learning and teaching: The past, current status and future directions. Front. Psychol. 12, 1–10. doi: 10.3389/fpsyg.2021.731721, PMID: 34489835 PMC8417049

[ref149] Wang X. Su Y. (2019). Revision of QCAE empathy scale for Chinese adolescents. Psychol. Tech. Appl. 7, 536–547. doi: 10.16842/j.cnki.issn2095-5588.2019.09.003

[ref150] Wen Z. Huang B. Tang D. (2018). Preliminary work for modeling questionnaire data. J. Psychol. Sci. 41, 204–210. doi: 10.16719/j.cnki.1671-6981.20180130

[ref151] Wu X. Xu H. Zhang X. Han S. Ge L. Li X.. (2021). Self-efficacy, Hope as mediators Between positive coping and resilience Among patients With gastric cancer Before the first chemotherapy. Cancer Nurs. 44, 79–85. doi: 10.1097/NCC.0000000000000753, PMID: 31743154

[ref152] Yalcin-Tilfarlioglu F. Arikan A. (2012). Empathy levels and academic achievement of foreign language learners. Procedia Soc. Behav. Sci. 46, 4428–4430. doi: 10.1016/j.sbspro.2012.06.268

[ref153] Yang Y. Xu X. Liu W. Pang W. (2020). Hope and creative self-efficacy as sequential mediators in the relationship Between family socioeconomic status and creativity. Front. Psychol. 11. doi: 10.3389/fpsyg.2020.00438, PMID: 32256427 PMC7090163

[ref154] Yin X. (2021). The interplay of EFL students’ enjoyment, Hope, pride and self-regulation. Front. Psychol. 12:803476. doi: 10.3389/fpsyg.2021.803476, PMID: 34938249 PMC8685263

[ref155] Youssef C. M. Luthans F. (2007). Positive organizational behavior in the workplace: The impact of Hope, optimism, and resilience. J. Manag. 33, 774–800. doi: 10.1177/0149206307305562

[ref156] Zeinalipour H. (2021). School connectedness, academic self-efficacy, and academic performance: mediating role of hope. Psychol. Rep.:10069. doi: 10.1177/00332941211006926, PMID: 33818192

[ref157] Zenasni F. Besancon M. Lubart T. (2008). Creativity and tolerance of ambiguity: An empirical study. J. Creat. Behav. 42, 61–73. doi: 10.1002/j.2162-6057.2008.tb01080.x

[ref158] Zhang C. (2020). “A study on academic emotional tendency of online learning for foreign language majors under the background of epidemic prevention and control.” in *2020 International Conference on Big Data and Informatization Education (ICBDIE)*; April 23–25, 2020; 346–349.

[ref159] Zhang Y. Liu W. Liu Y. Huang Z. Liu Q. (2019). Chinese college students’ optimism and social creativity mediated by creative self-efficacy and hope. Soc. Behav. Pers. 47, 1–9. doi: 10.2224/sbp.8268

[ref160] Zhou X. Wu X. Zhen R. (2017). Self-esteem and hope mediate the relations between social support and post-traumatic stress disorder and growth in adolescents following the Ya’an earthquake. Anxiety Stress Coping 31, 32–45. doi: 10.1080/10615806.2017.1374376, PMID: 28882068

[ref161] Zirenko M. Kornilova T. Qiuqi Z. Izmailova A. (2021). Personality regulation of decisions on physical distancing: cross-cultural comparison (Russia, Azerbaijan, China). Personal. Individ. Differ. 170:110418. doi: 10.1016/j.paid.2020.110418, PMID: 33041413 PMC7538945

