# Corrigendum: How does the optimism of students learning a foreign language affect their creative self-efficacy? The mediating effects of hope and empathy

**DOI:** 10.3389/fpsyg.2022.1023143

**Published:** 2022-09-23

**Authors:** Fei Lei, Lin Lei

**Affiliations:** ^1^School of Foreign Studies, South China Normal University, Guangzhou, China; ^2^Center for Language Cognition and Assessment, South China Normal University, Guangzhou, China; ^3^School of Management, Guangdong University of Technology, Guangzhou, China

**Keywords:** optimism, hope, empathy, creative self-efficacy, creativity, foreign-language learners, positive psychology

In the published article, the Funding statement was not included. The correct funding statement appears below:

## Funding

This work was supported by the National Social Science Fund of China.

The authors apologize for this error and state that this does not change the scientific conclusions of the article in any way. The original article has been updated.

## Publisher's note

All claims expressed in this article are solely those of the authors and do not necessarily represent those of their affiliated organizations, or those of the publisher, the editors and the reviewers. Any product that may be evaluated in this article, or claim that may be made by its manufacturer, is not guaranteed or endorsed by the publisher.

